# Cardiomyopathy phenotypes in human-induced pluripotent stem cell-derived cardiomyocytes—a systematic review

**DOI:** 10.1007/s00424-018-2214-0

**Published:** 2018-10-15

**Authors:** Thomas Eschenhagen, Lucie Carrier

**Affiliations:** 1 Institute of Experimental Pharmacology and Toxicology, University Medical Center Hamburg-Eppendorf, Hamburg, Germany; 2 Partner Site Hamburg/Kiel/Lübeck, DZHK (German Centre for Cardiovascular Research), Hamburg, Germany

**Keywords:** hiPSC, Disease modelling, Cardiomyopathy, Quantitative phenotypes

## Abstract

Human-induced pluripotent stem cells (hiPSC) can be differentiated to cardiomyocytes at high efficiency and are increasingly used to study cardiac disease in a human context. This review evaluated 38 studies on hypertrophic (HCM) and dilated cardiomyopathy (DCM) of different genetic causes asking to which extent published data allow the definition of an in vitro HCM/DCM hiPSC-CM phenotype. The data are put in context with the prevailing hypotheses on HCM/DCM dysfunction and pathophysiology. Relatively consistent findings in HCM not reported in DCM were larger cell size (156 ± 85%, *n* = 15), more nuclear localization of nuclear factor of activated T cells (NFAT; 175 ± 65%, *n* = 3), and higher β-myosin heavy chain gene expression levels (500 ± 547%, *n* = 8) than respective controls. Conversely, DCM lines showed consistently less force development than controls (47 ± 23%, *n* = 9), while HCM forces scattered without clear trend. Both HCM and DCM lines often showed sarcomere disorganization, higher *NPPA/NPPB* expression levels, and arrhythmic beating behaviour. The data have to be taken with the caveat that reporting frequencies of the various parameters (e.g. cell size, NFAT expression) differ widely between HCM and DCM lines, in which data scatter is large and that only 9/38 studies used isogenic controls. Taken together, the current data provide interesting suggestions for disease-specific phenotypes in HCM/DCM hiPSC-CM but indicate that the field is still in its early days. Systematic, quantitative comparisons and robust, high content assays are warranted to advance the field.

## Introduction

The seminal discovery of means to reprogram human somatic cells into embryonic stem cell-like induced pluripotent stem cells (hiPSC; [89]) opened the possibility to generate patient- and disease-specific hiPSC lines and study disease mechanisms in an individualized and human context. An underlying assumption is that human diseases can be studied in hiPSC-derived differentiated cells cultured in vitro or, in other words, that such cells exhibit disease-specific phenotypes. Indeed, soon after the discovery by Yamanaka and colleagues, the first papers appeared that reported specific abnormalities in the function of patient-derived hiPSC derivatives compared to unrelated genetically normal controls. In the cardiac field, the first examples were longer action potentials in hiPSC-cardiomyocytes (hiPSC-CM) from patients with genetically determined long QT syndrome 1 (LQT1 [68] or LQT2 [40]) and larger cells with a higher degree of sarcomeric organization and preferential localization of NFATc4 in the nucleus in hiPSC-CM from a patient with Leopard syndrome [11]. In the meantime, most genetically determined cardiac diseases have been studied in hiPSC-CM and generally revealed some phenotypic abnormalities that have been described before in native cardiomyocytes from patients with the respective disease. However, it was soon realized that hiPSC-CM are relatively immature cells (for review, see [105]) that exhibit large phenotypic heterogeneity, e.g. in terms of action potential width and shape [68], cell size and sarcomeric organization. Reasons include variability of the original somatic cells used for reprogramming (e.g. skin cells with mosaic mutations or variable levels of epigenetic modifications [53], the reprogramming procedure itself [46], differentiation protocols with less than 100% efficiency, a varying level of maturity in hiPSC-CM in culture as well as methodological issues such as the difficulty to measure action potentials in small cells by patch clamping [38]). The recent introduction of transcription activator-like effector nuclease-mediated gene correction (TALEN) or CRIPSR/Cas9-based methods for gene editing has increased the level of trust in the conclusion that the observed phenotypes were indeed the consequence of the suspected gene mutation [4]. The reader is referred to several excellent reviews on this subject (e.g. [8, 70, 107]).

## Hypertrophic and dilated cardiomyopathy—clinical phenotype and pathophysiology

This review will concentrate on the question to which extent a specific “cardiomyopathy phenotype” exists, which can be studied in hiPSC-CM in the dish. It restricts itself to hypertrophic cardiomyopathy (HCM) and dilated cardiomyopathy (DCM), because they are the two most common and clinically relevant cardiomyopathies, often have a defined genetic cause, have been most often studied in hiPSC-CM and present with relatively clearly defined and partially opposing clinical phenotypes (Table 1). The key morphological features of HCM are thickened left ventricular (LV) walls in the absence of apparent hemodynamic reason (e.g. aortic stenosis, severe hypertension). Hypertrophy preferentially affects the interventricular septum, whose thickness is commonly used as inclusion criterion for patients in clinical studies. HCM is generally associated with a normal or rather small LV cavity, preserved LV systolic contractile function and early diastolic dysfunction [37, 66, 102]. Most patients develop various degrees of LV obstruction [60]. Histomorphological signs of HCM are myocardial disarray and increased fibrosis. DCM in contrast is characterized by LV systolic dysfunction, dilation of LV cavities and normal wall thickness. While HCM is the prototypic genetic cardiomyopathy (likely disease-causing mutations can be found by cardiac gene panel, exome or whole genome sequencing in approximately 32–70% of cases [2, 16, 58, 78]), DCM is classified as a mixed cardiomyopathy, which is familial in ~ 20–35% [25, 60], and a recent whole exome sequencing identified mutations in only 12% of cases [58]. The majority of DCM cases are caused by (mainly viral or parasitic) infection, toxins such as alcohol or anti-tumour agents and mitochondrial disorders.

**Table 1:** Clinical, morphological and functional characteristics of patients with hypertrophic (HCM) or dilated cardiomyopathy (DCM)

	HCM	DCM
Symptoms and biomarkers	Arrhythmias and sudden cardiac death	Dyspnoe (initially exercise-induced)
	Atrial fibrillation	Heart failure
	Exercise-induced dyspnoe	Arrhythmias and sudden cardiac death
	Heart failure	Atrial fibrillation
	Increased serum BNP levels	Increased serum BNP levels
Morphology	*LV hypertrophy ± outflow tract obstruction*	*LV chamber dilatation*
	Cardiac myocyte hypertrophy (width)	Cardiac myocyte hypertrophy (length)
	*Myofiber/myocardial disarray*	
	Fibrosis	Fibrosis
Function	Diastolic dysfunction (pre-hypertrophy stage)	*Systolic dysfunction*
	Hypercontractility (inconsistent)	Diastolic dysfunction
	Systolic dysfunction (late stage)	
	Energy depletion (early stage)	Energy depletion (early stage)

Parameters distinguishing between HCM and DCM are marked in italics. Note overlap of many parameters

While the partially opposing clinical pictures of HCM and DCM allow a relatively straightforward clinical differentiation, overlaps between the two types of cardiomyopathies exist. Both HCM and DCM exhibit increased serum levels of brain natriuretic peptide and cardiac fibrosis, and HCM patients can develop severe systolic dysfunction requiring heart transplantation. Both can lead to life-threatening ventricular arrhythmias [60] and are accompanied by an increased rate of atrial fibrillation [81, 106] and dilation of the left atrium [36].

Despite the discovery of numerous mutations in genes that underlie HCM and DCM, our understanding of the pathomechanisms leading from the mutation to the phenotype remains incomplete. Reasons are not only the diversity of mutations causing similar clinical pictures, particularly in DCM, the incomplete and highly variable penetrance of both HCM and DCM, but also the fact that mouse models only partially recapitulate the human phenotype. For example, no single mouse model in which a classical HCM mutation in the gene coding for cardiac myosin-binding protein C (*MYBPC3*, cMyBPC) or β-myosin heavy chain (*MYH7*, β-MHC) has been introduced in the heterozygous state develop the pathognomonic septal hypertrophy seen in patients (for review, see [21]). Neither has LV obstruction been observed in any such model. Either homozygous knockout or knockin of the respective gene is lethal (as in the case of α-MHC, the rodent pendant of the dominant myosin isoform [28]) or the animals develop severe LV dysfunction (as in the case of cMyBPC [10, 27, 33, 61, 62, 64, 65, 98]). It is also apparent that the mouse work still did not answer a number of fundamental questions: (1) What is the exact physiological role of the sarcomeric proteins most commonly affected in HCM such as cMyBPC and β-MHC? (2) How do they cooperate to ensure proper systolic and diastolic function? (3) What are the specific consequences of even relatively well-studied gene mutations? (4) How do mutations in numerous sarcomeric and non-sarcomeric genes with diverse function lead to the uniform induction of “autonomous” cardiac hypertrophy and disarray in HCM? These questions have been discussed in recent reviews to which the reader is referred [21, 26, 59, 94]. In any case, the experiences with mouse models thus raise the question to which extent they really reflect the human disease and provide an argument to study HCM and DCM in hiPSC-CM.

Another reason for our limited understanding of HCM/DCM pathophysiology is that access to isolated heart tissue and cells from patients with HCM and DCM is sparse, and only very few studies specifically reported on the in vitro phenotype of these diseases (for review, see [22]). The most commonly used sources for human tissues are septum biopsies acquired during surgical correction of LV outflow tract obstruction by myectomy in the case of HCM and LV tissues obtained during implantation of LV assist devices or heart transplantation in case of DCM. Both tissue sources represent a late stage of the disease, raising the question to which extent the abnormalities observed in comparison to (even rarer) non-failing heart tissue comparators reflect primary defects or secondary compensations or consequences. The highly fibrotic texture of the terminally diseased tissue imposes a further challenge to such studies as enzymatic isolation of cardiomyocytes requires harsher conditions, introducing a systematic error.

## Prevailing in vitro phenotypes of HCM and DCM

Despite the limitations discussed above, some observations prevail and have led to hypotheses that can be tested in hiPSC-CM.

### Abnormal myofilament calcium sensitivity

The relation between intracellular Ca^2+^ concentrations and force development of the myofilaments is a highly regulated biological constant with half-maximal force development (of skinned myofibers) at a pCa of ~ 5.8 (~ 1.6 μM). Numerous studies reported increased Ca^2+^ sensitivity on HCM [3, 13, 19, 23, 67, 95, 96] and decreased in DCM [19, 20, 56]. The shift in the pCa/force relation leads to more force development at lower Ca^2+^ concentrations in case of HCM and less force development in DCM. Importantly, the increased Ca^2+^ sensitivity in HCM also predicts delayed relaxation in the descending part of the intracellular Ca^2+^ transient. Both consequences are well compatible with the predominant clinical phenotypes of preserved systolic function and diastolic dysfunction in HCM and systolic dysfunction in DCM. Increased Ca^2+^ sensitivity in HCM would even predict LV hypercontractility at rest, and indeed, a study in 36 mutation carriers found significantly increased LV ejection fraction by echocardiography compared to 36 healthy controls [36]. This observation forms the basis of novel therapeutic concepts to reduce myosin activity by small molecules to treat HCM [30]. However, it is also clear that increased Ca^2+^ sensitivity in HCM is not a universal finding. Several studies reported HCM mutations to be associated with either no change [33, 101] or decreased Ca^2+^ sensitivity [88] in various experimental systems, suggesting mutation-specific differences. Of note, even the knockout of a protein such as cMyBPC [13, 33, 48] has been associated with different effects on myofilament Ca^2+^ sensitivity. The latter emphasizes the importance of the respective experimental context and supports the notion that altered myofilament Ca^2+^ sensitivity cannot be the sole unifying mechanism underlying HCM or DCM.

### Abnormal actin-myosin sliding velocity

Another parameter of sarcomere function is the unloaded sliding velocity of thin filaments on immobilized S1-myosin. Several studies indicate that HCM mutations are associated with increased sliding velocity [44, 45, 88] and DCM with decreased sliding velocity [1, 79], and for a review, see [24].

### Altered maximal force development

Interestingly, both HCM and DCM mutations were found to generally associate with decreased maximal force development [95, 101], but normal or even higher force output and increased force redevelopment have also been reported [48, 92, 102].

### Increased Ca^2+^-independent cross-bridge cycling in HCM

Mutations (or full deletion) of *MYBPC3* or cardiac troponin T (*TNNT2*, TnT) have been associated with a shallow pCa-force relationship (lower Hill coefficient) and residual force development at very low or nominal absence of Ca^2+^ [3, 56, 75]. In the case of cMyBPC, the effect may be explained by mutations (or its absence) disturbing its normal role in stabilizing the super-relaxed, inactive state (SRX) of myosin heads [63]. The concept implies that one of the abnormalities in HCM is incomplete arrest of crossbridge cycling in diastole, which could participate in diastolic dysfunction and increased energy expenditure.

### Decreased energetic efficiency

Many HCM mutations lead to decreased energetic efficiency of crossbridge cycling, i.e. inefficient usage of ATP to fuel contraction [14, 45, 67, 102]. The phenomenon in a general sense indicates less-than-normal functioning of the sarcomeres harbouring mutated sarcomere proteins and may relate to the partial loss of the myosin SRX state in the case of cMyBPC. In any case, it may well contribute to the decreased phosphocreatine/ATP ratio observed in patients with HCM even in the prehypertrophic state [17]. Energy starvation is not specific to HCM. In fact, it has been shown already in 1992 as a common feature of patients with heart failure due to non-ischemic DCM [71]. However, it is possible that the more diverse causes of DCM include both decreased energetic efficiency of the myofilaments with higher energy expenditure and decreased efficiency of mitochondrial energy generation like in Barth syndrome [39].

### Allelic imbalance of β-MHC as a cause of myocardial disarray

Early work (in skeletal muscle fibres from β-MHC-expressing soleus muscle) showed a high variability of myofilament Ca^2+^ sensitivity between individual muscle fibres [47]. This observation was later related to marked cell-to-cell differences in the expression of the mutated β-MHC in cardiomyocytes and marked differences in individual Ca^2+^ sensitivity [49]. The interesting phenomenon could well contribute to another hallmark of HCM, myocardial disarray, by individual cardiomyocyte developing different degrees of contractile force. It is not clear whether allelic imbalance is restricted to β-MHC mutations.

## HCM and DCM phenotypes in hiPSC-cardiomyocytes

By searching PubMed (keywords: *hiPSC cardiomyocytes* and *hypertrophic cardiomyopathy* or *dilated cardiomyopathy*), we identified 38 original papers reporting phenotypes of hiPSC-CM either derived from hiPSC lines of patients with HCM/DCM (or related syndromes) or from hiPSC lines in which a HCM or DCM mutation had been genetically introduced (Tables 2 and 3). Initial studies compared the phenotype of disease-related hiPSC-CM to unrelated genetically healthy controls; more recent studies used TALEN or CRISPR/Cas9 gene editing approaches to correct a mutation in a patient-specific line or introduce it into a wild-type line, allowing comparison under isogenic conditions. While most studies validated the absence of off-target effects only at the predicted top-10 sites, one TALEN-based study performed whole exome sequencing and reported in two corrected clones 318 and 1331 de-novo indel mutations, respectively, close to possible off-target sites. The significance of this finding is unclear.

**Table 2:** Studies reporting cardiomyocyte phenotypes in hiPSC-CM from patients with genetically determined HCM or syndromes associated with HCM phenotypes or from hiPSC lines in which mutations had been introduced

Mutation	Disease	Peak force	T1	T2	Cell size	Disarray	Other phenotypes	CRISPR/TALEN Ctr.	Karyotype Ctr.	Reference
*MYH7* Het p.Arg663His	HCM	n.d.	n.d.	n.d.	+ 60%	n.d.	Multinucleation 50 vs. 20%, mRNA of *NPPA*, *NPPB*, *MYH7* up, higher nuclear NFAT, Ca^2+^-induced arrhythmias, DAD, higher basal [Ca^2+^], Iso-induced DAD	No	No	[50]
*MYBPC3* 3 Pts, 3 Ctr p.Gly999-Gln1004del, 2 w/o identified mut	HCM	n.d.	n.d.	n.d.	+ 20%	+ 50–100%	*NPPA*, *TNNT2* up, *MYBPC3*–20% in mut, stronger hypertrophic, disarray and NFAT response to ET1, contractile abnormalities linked to disarray	No	No	[90]
*MYBPC3* Exon 25, 3 pts.	HCM	n.d.	n.d.	n.d.	+ 50–100%	n.d.	No further hypertrophic response to stimuli	No	Yes	[18]
*MYH7* Het p.Arg442Gly	HCM	n.d.	n.d.	n.d.	+ 15%	+ 200%	Higher nuclear NFAT; arrhythmias + 300%, APD prolongation + 60%, resting [Ca^2+^] up 20%, I_Ca_ and I_Na_ up	No	Yes	[32]
*MYBPC3* Het c.2373dupG	HCM	− 50%	n.d.	n.d.	+/−	n.d.	cMyBPC haploinsufficiency	No	No	[7]
*GAA* Hom del in exon 18 and CpHet c.1441delT/c.2237G>A 2 lines each	Pompe	+/− or − 60%	+/−	+/−	n.d.	n.d.	Glycogen accumulation, glycan processing abnormality, but normal autophagic flux	No	Yes	[77]
GAA CpHet c.796C>T/ c.1316T>A, 3 clones from pt., 1 clone from ctr	Pompe	n.d.	n.d.	n.d.	no	n.d.	Glycogen accumulation, no functional data	No	No	[82]
*ALPK3* Hom p.W1264X	DCM/HCM	n.d.	n.d.	n.d.	n.d.	+ 230%	Irregular Ca^2+^ transients + 400%, MEA FP + 100%	No	Yes	[73]
*BRAF* Het p.Thr599Arg	HCM Syndromic	0 to + 40%	− 30%	− 30%	n.d.	n.d.	Less negative FFR, increased Iso-sensitivity, mRNA of *NPPA* + 300% and *SERCA2a* + 40% (ns)	No	No	[12]
*BRAF* Het p.Thr599Arg Het p.Gln257Arg	HCM Syndromic	n.d.	n.d.	n.d.	+ 300%	+ 260%	mRNA of *NPPA*, *NPPB*, *MYH7* up, *PLN* down, higher Ca^2+^ transients and store, calcium arrhythmias, “fibroblast” profibrotic phenotype	No	Yes	[41]
*FXN* GAA triplet repeat	Friedreich’s ataxia	n.d.	n.d.	n.d.	n.d.	n.d.	ROS, unusual iron responses	No	No	[51]
*MYBPC3* Het p.Gln1061X (*n* = 2) or *TPM1* Het p.Asp175Asn (*n* = 2)	HCM	n.d.	n.d.	n.d.	+ 200% (M), not clear in T	n.d.	More multinucleation (40 vs 20%), Ca^2+^ arrhythmias (T, not M), more DAD in M, not T, APD high in T, RMP − 75, *NPPA* +/−, *NPPB*, *MYH7* and many others up, more in M than T, cMyBPC +/−, TPM up in T	No	Yes	[72]
*MYH7* Het p.Glu848Gly	HCM	− 54%	n.d.	n.d.	n.d.	Yes, not quantified	Skinned myofiber from hiPSC-CM: Fmax 8.2 vs. 18.6 mN/mm^2^ (adult 110), KAct + 62%, increased Ca^2+^ sensitivity	No	No	[74]
*MYH7* Het p.Val698Ala	HCM	n.d.	n.d.	n.d.	n.d.	n.d.	n.d.	N.d.	Yes	[80]
*GLA* Hemizygote c.919+4G >A	Fabry	n.d.	n.d.	n.d.	n.d.	n.d.	Gal act. down, GB3 accumulation, low beating rate, arrhythmias	No		[15]
*LAMP2* *Het c.129–* *130insAT)* *Het c.64+1G>A*	Danon	n.d.	n.d.	n.d.	n.d.	n.d.	Mitochondrial abnormalities, decreased autophagic flux	No	Yes	[34]
*MYBPC3* Het c.1358_1359insC	HCM	n.d.	n.d.	n.d.	+ 65%	n.d.	cMyBPC haploinsufficiency, BNP, *MYH7* and others up	Corrected by gene therapy	No	[76]
*PRKAG2* Het Arg302Gln	HCM + WPW	n.d.	n.d.	n.d.	+ 10–30%	n.d.	MDP, APD +/−, If +/−, AP irregularity, RR scatter + 500%	Yes	Yes	[5]
*SCO2* Hom c.577G>A CpHet c.418G>A/c.17Ins19	HCM syndrome	+/− (??)	n.d.	n.d.	n.d.	n.d.	Mitochondrial abnormalities, no Iso or Ca^2+^ response, DAD, arrhythmic response to Iso	No	Yes	[31]
*MT-RNR2* m.2336T>C	Mitochondrial HCM	n.d.	n.d.	n.d.	+ 30%	n.d.	*NPPA*, *NPPB*, *NFAT* up, slightly increased intracellular calcium, SR store, reduced I_Ca_, APD prolonged, arrhythmias, RMP − 55, upstroke 5–10 v/s, DAD	No	Yes	[52]
*MYL3* Het c.170C-A, Exac 0.0001154, introduced 170C-g and *MYBPC3* Het p.Val321Met	HCM-associated VUS	n.d.	n.d.	n.d.	+/− (also in mut)	n.d.	No phenotype detected in VUS, mean cell size 1800 μm^2^, *NPPA* and *MYH7* up in the two diseased, contraction and rel velocity slightly up, arrhythmias, good stats	Yes	Yes	[57]
*TNNT2* Het p.Ile79Asn	HCM	+ 75%	n.d.	+ 40%	+/−	yes	Sarcomere length +/− (1.8 μm), smaller caffeine transient, higher Ca^2+^ buffering, shorter APD, Ca^2+^ beat to beat instability, triangulation, NCX-sensitive	Yes	Yes	[100]
*MYH7* and *MYH6*; Het/Hom p.Arg453Cys, frameshift KO, +*MYH6* frameshift	HCM	− 20% (het), − 70% (hom) − 80% (KO)	+ 20%	+/− or + 10% (+*MYH6* fs)	+ 50%	yes	*NPPB* up, multinucleation, basal and max. respiration up, ATP production up, Ca^2+^ transient irregularities, nifedipine- and ranolazine-sensitive; *MYH7/MYH6* ratio up	Yes	Yes	[69]

*ANP/BNP* atrial/brain natriuretic peptides, *AP* action potential, *APD* action potential duration, *ALPK3* α-kinase 3, *BRAF* B-Raf proto-oncogene, serine/threonine kinase, *cMyBPC* cardiac myosin-binding protein C, *CpHet* compound heterozygous, *CRISPR* clustered regularly interspaced short palindromic repeats/Cas9 gene correction, *Ctr* control, *DAD* delayed after depolarizations, *Del* deletion, *Disarray* abnormal sarcomeric organization, *ET1* endothelin 1, *FFR* force-frequency relation, *Fmax* maximal force development, *FXN* frataxin gene, *GAA* α-glycosidase gene, *GLA* α-galactosidase A, *GB3* glycosphingolipids, *Het* heterozygous, *Hom* homozygous, *I*_*Ca*_ L-type Ca^2+^ current, *I*_*Na*_ Na^+^ current, *Iso* isoprenaline, *KAct* rate constant reflecting crossbridge turnover rate, *LAMP2* lysosome-associated membrane protein 2 gene, *MEA FP* multielectrode array field potentials, *MT-RNR2* mitochondrially encoded 16S RNA gene, *Mut* mutation, *MYBPC3* cardiac myosin-binding protein C gene/mRNA, *MYL3* myosin light chain 3 (MLC1v) gene, *MYH6/MYH7* gene or mRNA of α-/β-myosin heavy chain, *NCX* sodium-calcium exchanger, *NFAT* nuclear factor of activated T cells, *NPPA* atrial natriuretic peptide mRNA, *NPPB* brain natriuretic peptide mRNA, *PLN* phospholamban gene/mRNA, *Pt(s)* patient(s), *RMP* resting membrane potential, *ROS* reactive oxygen species, *RR* scatter beat-to-beat irregularity, *SERCA2a* sarcoplasmic reticulum ATPase, *SCO2* cytochrome c oxidase assembly protein gene, *T1* time to peak force, *T2* time from peak to relaxation, *TALEN* transcription activator-like effector nuclease-mediated gene correction, *TNNT2* cardiac troponin T gene, *TnT* cardiac troponin T, *TPM1* α-tropomyosin, *VUS* variant of unknown significance

**Table 3:** Studies reporting cardiomyocyte phenotypes in hiPSC-CM from patients with genetically determined DCM or syndromes associated with DCM phenotypes or from hiPSC lines in which mutations had been introduced

Mutation	Disease	Peak force	T1	T2	Cell size	Disarray	Other phenotypes	CRISPR/TALEN Ctr.	Karyotype Ctr.	Reference
*TNNT2* Het p.Arg173Trp	DCM	− 80% (AFM)	n.d.	n.d.	+/−	Yes	Desensitized NE response of rate, RMP − 40 mV, APD +/−, smaller Ca^2+^ and caffeine transient (~2 s), TTP and TTD caffeine + 100% (WT 0.5/1.2 s), rescue by SERCA OE, metoprolol rescues disorganization	No	Yes	[87]
*LMNA* Het p.Arg225X (*n* = 3), another fs mut	DCM	n.d.	n.d.	n.d.	n.d.	n.d.	Nuclear abnormalities, apoptosis,MEK-inhibition-sensitive, ERK up	No	Yes	[84]
*DES* Het p.Ala285Val, + 43 stop/gain mut	DCM	n.d.	n.d.	n.d.	n.d.	n.d.	Morphological abnormalities + 700%, aggregates, peak Ca^2+^ transient +/−, − dF/dt − 40%, abnormal Iso response	No	No	[91]
*TAZ* Het c.517delG Het c.328T>C	Barth	− 50–70%	n.d.	n.d.	n.d.	Yes	Decreased mito ox rate, immature cardiolipin, reduced ATP content, excess ROS, tafazzin-sensitive, MitoTempo-sensitive	Yes	Yes	[99]
*TNNT2* p.Arg173Trp	DCM	− 60% (TFM)	n.d.	n.d.	n.d.	n.d.	ISO response down, TTP/TTD +/− (~1 s), cAMP response to Iso − 50%, rate − 60%, PDE2,3,5 mRNA several folds up, rescue of cAMP and force by FSK + IBMX	No	No	[103]
*TTN* p.Ser14450fsX4	DCM	n.d.	n.d.	n.d.	n.d.	Yes	*MYH6*, *MYH7*, *ACTC1*–50%, rescue by exon skipping	No	No	[29]
*DMD* Dp427m	DMD	n.d.	n.d.	n.d.	n.d.	n.d.	More apoptosis, almost complete lack of *MYL2*, *MYL3* and *TPM1* mRNA, I_Ca_ − 40%, resting [Ca^2+^] + 40%, beneficial effects by Poloxamer 188		No	[54]
*TTN* 3 diff. Truncating mut, CRISPR induction	DCM	− 60–80% (EHT)	n.d.	n.d.	n.d.	+ 400%	Stronger phenotype on stiffer posts, rate − 50%, lower SL (1.6 vs. 1,75 μm), lower *MYH7/MYH6* ratio, beneficial effect of VEGF	No	No	[35]
*PLN* Het p.Arg14del (R14del)	DCM	− 60–90%	n.d.	up	n.d.	n.d.	Resting [Ca^2+^] + 30%, irregular Ca^2+^ transient + 500%, caffeine transient + 75%, RMP − 49 vs. − 58 mV, *NPPA/NPPB* + 300–900%, *MYH7/MYH6* + 60%	Off-target effects	Yes	[43]
*RBM20* p.Arg636Ser 2 clones each WT/mut	DCM	n.d.	n.d.	n.d.	n.d.	Yes	Increased sarcomere length (WT 1.4 μm), increased Ca^2+^ peak, AUC, decay time at much lower rate		Yes	[104]
*TNNT2* p.Arg173Trp 2 clones each	DCM	(−) Inconsistent	n.d.	n.d.	n.d.	Yes	Lower rate of cells beat, myosin ATPase − 20%, beneficial effects of omecamtiv		No	[9]
*RBM20* p.Ser635Ala 2 clones each	DCM	− 40% (EHT)			+/− (100 μm^2^)	Yes	Actinin periodicity − 80%, lower resting [Ca^2+^], peak [Ca^2+^] + 50%, TTP/TTD + 60/150%, duration + 150%, all at 70% lower rate, normal Iso force response, lower length-stress response, *TTN* + *MYH7* exon exclusion		No	[86]
*BAG3* KO induced in WT, 2 lines *MYBPC3* KO	DCM	− 50% (MTF)	n.d.	n.d.	n.d.	Yes	Stronger disarray increased by bortezomib, normal bortezomib-response in *MYBPC3*-KO	Yes	Yes	[42]
*DMD* 3 different mut	DMD	− 30–70% (EHT)	n.d.	n.d.	n.d.	n.d.	Correction of phenotype by CRISPR	Yes	No	[55]
*DMPK* CTG repeats in, 4 × 6 clones analysed	DM1	Rundown	n.d.	n.d.	n.d.	n.d.	Nuclear RNA CUG foci, nuclear size + 30%, irregularity, MDP − 52 vs. − 60 mV, APD − 20%, AP amplitude − 20%, upstroke velocity down, force rundown (AFM); altered splicing of *MBNL1*, *MBNL2*, *TNNT2*, *SCN5A*; fetal *SCN5A* isoforms, α-MHC and TnT protein down	No	Yes	[85]

*ACTC1* α-cardiac actin gene, *α-MHC* α-myosin heavy chain, *AFM* atomic force microscopy, *ANP/BNP* atrial/brain natriuretic peptide, *AP* action potential, *APD* action potential duration, *AUC* area under the curve, *BAG3* BCL2-associated athanogene 3, *cAMP* cyclic adenosine monophosphate, *CRISPR* clustered regularly interspaced short palindromic repeats/Cas 9 gene correction, *DES* desmin gene, *Disarray* abnormal sarcomeric organization, *DM1* myotonic dystrophy type 1, *DMD* Duchenne muscular dystrophy, *DMD* dystrophin gene, *DMPK* dystrophia myotonica protein kinase, *EHT* engineered heart tissue, *ERK* extracellular signal-regulated kinase, an important MAPK, *Fs* frameshift, *FSK* forskolin, *IBMX* isobutylmethylxanthine, *I*_*Ca*_ L-type Ca^2+^ current, *Iso* isoprenaline, *KO* knockout, *LMNA* lamin A/C gene, *MBNL* muscle blind-like, *MDP* maximal diastolic potential, *MEK* mitogen-activated protein (MAPK) kinase kinase, *Mut* mutation, *MYBPC3* cardiac myosin-binding protein C (cMyBPC) gene, *MYH6*/*MYH7* mRNA of α-/β-myosin heavy chain, *MYL2* regulatory myosin light chain gene, ventricular isoform (MLC2v), *MYL3* essential myosin light chain gene (MLC1v), *NPPA* atrial natriuretic peptide gene/mRNA, *NPPB* brain natriuretic peptide gene/mRNA, *NE* norepinephrine, *PDE* phosphodiesterase, *PLN* phospholamban gene, *RBM20* RNA binding motif protein 20 gene, *RMP* resting membrane potential, *ROS* reactive oxygen species, *SCN5A* sodium voltage-gated channel alpha subunit 5 (Nav1.5) gene, *SERCA OE* sarcoplasmic reticulum ATPase overexpression, *SL* sarcomere length, *T1* time to peak force, *T2* time from peak to relaxation, *TALEN* transcription activator-like effector nuclease-mediated gene correction, *TAZ* tafazzin gene, *TFM* traction force microscopy, *TNNT2* cardiac troponin T gene/mRNA, *TnT* cardiac troponin T, *TPM1* tropomyosin gene, *TTD* time-to-decay, *TTN* titin gene, *TTP* time-to-peak, *VEGF* vascular endothelial growth factor, *WT* wild type

The initial analysis of the papers concentrated on abnormalities in contractile function, based on the hypothesis formulated by Davis and Molkentin that differences between HCM, DCM and wild type (WT) should primarily result in a different tension-time integral of the contraction peak, i.e. the area under the curve of an averaged contraction peak [19]. The hypothesis corroborates the idea that HCM mutations lead to increased, DCM mutations to decreased myofilament Ca^2+^ sensitivity. As elegantly shown in mouse models with different cardiac troponin C mutations (and in examples of hiPSC-CM), this should lead to higher peak force and prolonged relaxation (T2) in case of HCM and lower peak force and an abbreviated contraction peak (both contraction [T1] and relaxation time [T2]). Unfortunately, contraction kinetics were only studied in a small minority of cases (Tables 2 and 3). Only two studies of a DCM mutation (heterozygous phospholamban (*PLN*) R14del and truncating titin (*TTN*) mutation) showed a representative contraction peak, which indicated prolonged relaxation in one case [43] and lower T1 and T2 in the other [35]. Statistics were not provided. Two papers on HCM mutations (*TNNT2*, *MYH7*) reported statistically evaluated data on T1 and T2, showing no alteration or the expected increase in time of relaxation [69, 100]. Thus, clearly, more work has to be done to decide whether or not HCM/DCM mutations have a systematic effect on contractile kinetics in hiPSC-CM.

**Fig. 1:**
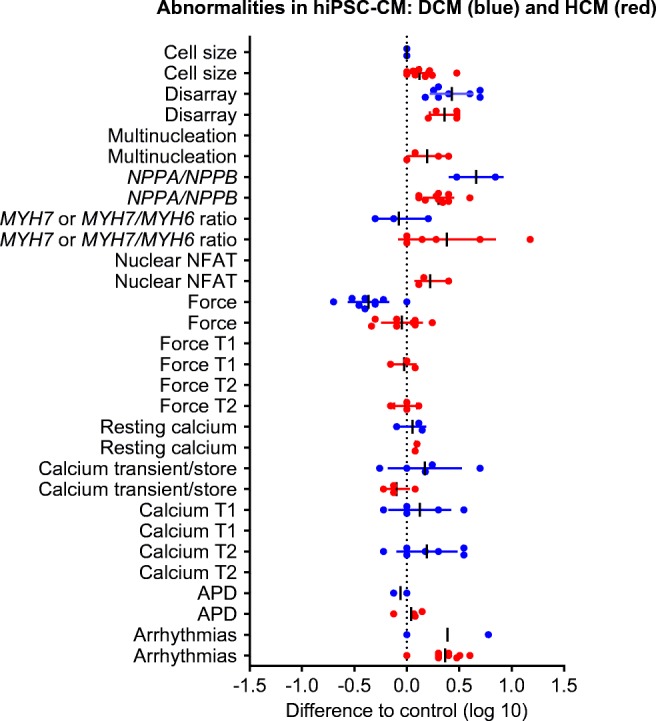
Published abnormalities of structure, gene expression or function of HCM/DCM-derived hiPSC-CM (HCM in red, DCM in blue). The data were extracted from the studies summarized in the Tables and are expressed as fold (log scale) of the control used in the respective study (either healthy control or gene edited isogenic line). More detail is provided in Tables 1 and 2. Each dot indicates one study. Lack of dots for certain parameters (e.g. force T1/T2 for DCM) indicates that none of the studies has reported these parameters. Abbreviations used: NPPA/NPPB atrial/brain natriuretic peptide (mRNA or protein concentration/positivity), MYH7/MYH6 β/α-myosin heavy chain gene expression, T1 time to peak of contraction or (calcium T1) of calcium transient peak, T2 time from contraction peak or (calcium T2) of calcium transient peak to relaxation/decay, APD action potential duration. The line calcium transient/store combines data on the peak calcium transient under baseline or caffeine-induced conditions

Many studies reported peak force, size of intracellular Ca^2+^ transients, sarcomere structure and gene expression (Tables 2 and 3, Fig. 1). Interestingly, while almost all studies on HCM lines reported cell sizes in 2D culture, only two did in case of DCM lines (Fig. 1). Similar differences in reporting frequency were observed with regard to multinucleation, nuclear NFAT, contraction kinetics (only HCM), ANP/BNP (*NPPA*/*NPPB*) and rhythmicity (more in HCM) or Ca^2+^ transient kinetics (only DCM). Reasons are unknown, but a reporting bias appears likely.

Figure 1 summarizes the data from all studies in which functional data were reported in a quantitative manner and presents them compared to the respective controls (log scale; *n* = 16 HCM, 14 DCM). Three abnormalities appeared to be relatively consistent in both HCM and DCM—sarcomeric disarray (274 ± 81%, *n* = 6 HCM; 298 ± 146%, *n* = 8 DCM), increased *NPPA* or *NPPB* gene expression (284 ± 249%, *n* = 11 HCM; 500%, *n* = 2) and arrhythmic behaviour (327 ± 164%, *n* = 12 HCM; 350%, *n* = 2 DCM). HCM lines showed an increase in cell size (156 ± 85%, *n* = 15; DCM +/−), in *MYH7* gene expression (or the ratio of *MYH7/MYH6* (500 ± 547%, *n* = 8; DCM +/− or reduction) and nuclear accumulation of the transcription factor NFAT (175 ± 65%, *n* = 3; DCM not determined). The most consistent abnormality in DCM lines was lower peak force development compared to the respective control (47 ± 23%, *n* = 9; HCM +/− with variability).

Besides the reported disease-associated abnormalities in function, structure or gene expression, it is apparent that absolute values varied largely. For example, reported cell surface area in 2D ranged from 100 μm^2^ [86] to > 2000 [57, 76], with reported cell volumes from 5.8 [100] to 120 μm^3^ [69]. Both volume data appear extremely low compared to the 95 μm^3^ in erythrocytes (mean corpuscular volume; Wikipedia). Besides differences in methods (e.g. time of culture in 2D, surface patterning), issues with the imaging technique and calculations may explain the scatter. In any case, hiPSC-CM are largely smaller than their native adult counterparts for which volumes of 15,000–40,000 μm^3^ have been reported [6]. It is not quite clear why size comparisons by patch clamp (membrane capacitance) indicate much smaller differences between hiPSC-CM and native human atrial or ventricular cardiomyocytes (e.g. 31–47 pF in hiPSC-CM compared to 74/89 pF in right atrial/LV myocytes [38]). The capacitance data are consistent across different studies (e.g. 60 pF in hiPSC-CM [93], 27 pF in hiPSC-CM [52, 54], ~ 60 pF in human atrial cardiomyocytes [97]). Possibly, the ratio between membrane capacitance and cell volume, which varies between species and the developmental stage (pF/pl = 4–9 [83]), is unusually high in hiPSC-CM. Action potential duration (APD90) at 37 °C varied from 240 ms [5] to 710 ms [43]. Again, it is likely that not only biological differences between hiPSC lines and influences of cell culture conditions and CM maturity but also technical issues explain the large variation. We have shown recently that the sharp microelectrode technique provides more reliable action potential data than patch clamping of single cells [38]. In this study, patch clamp-recorded APD90 in isolated hiPSC-CM amounted only to 119 ± 17 ms (human atrial cardiomyocytes 220 ms, human LV cardiomyocytes 434 ms), while those in intact hiPSC-CM or 3D engineered heart tissue were 271 ms (human right atrial tissue 317 ms, LV tissue 334 ms).

## Conclusion

The present overview on published reports on the phenotype of HCM/DCM-derived hiPSC-CM allows some preliminary conclusions. (1) The most consistent and to a certain degree differentiating phenotype of hiPSC-CM appears to be decreased force production in DCM, correlating well with the dominant clinical presentation of the disease. (2) HCM lines appear not to exhibit consistent alterations in force development but show increased CM size, nuclear NFAT and increased *MYH7* or *MYH7/MYH6* ratio. Given the paucity of measurements of these parameters in DCM, it is not possible at present to decide whether these parameters allow a distinction between HCM/DCM phenotypes. (3) Sarcomere disorganization is a common finding in all disease lines and does not appear to allow differentiation between the clinically opposing phenotypes. (4) Overall, the analysis indicates that hiPSC-based disease modelling of cardiomyopathies is still in its early days. Suggestions for a basal set of parameters to be analysed in future studies are given in Table 4. More statistical rigor and robust high content methods are necessary to uncover potentially meaningful but discrete abnormalities of cardiac function in these cells. In this respect, it is interesting to note that only one study evaluated myofilament Ca^2+^ sensitivity in skinned fibres [74], yet myofilament Ca^2+^ sensitivity is one of the most commonly studied parameters in HCM/DCM-related human or animal specimens.

**Table 4:** Suggestions for a basal set of parameters to be analyzed and reported in hiPSC-CM studies

Parameter	Comment
Karyotype	Karyotype problems are frequent and increase with passage number. Karyotype checks in iPSC should be done < 5–10 passages before analysis.
Cardiomyocyte yield	The percent of TnT- or actinin-positive cells (e.g. by FACS) per batch evaluated should be presented as mean ± SD.
Number of batches	The number of cells/derivatives (*n* = *x*) and the number of differentiation runs the cells were derived from (*N* = *y*) in a given experiment need to be reported.
Blinding procedures	Given the variability of cells and readouts, procedures should be established and described that allow investigator-blinded assessments.
Age of cardiomyocytes	Many parameters change over time of culture in 2D or 3D, therefore the age of cells at time of analysis should be presented (mean ± SD).
Expression of disease gene alleles	In case of defined mutations, the relative expression of mutant and wild-type alleles should be determined to get an idea of mechanism.
Gene expression	Transcript levels should be reported as a set of standard genes, not only selected examples.
Indicators of cardiomyocyte maturity	Absolute transcript levels of α-/β-MHC (+their ratio) in comparison with human heart levels give a good initial indication of maturity.
Cell size	High n-numbers and information on cell density are mandatory. Volume data (e.g. FACS) may be more informative and precise than surface measurements in 2D.
Force and force kinetics	Given the strong dependence of force and force kinetics on beating rate, temperature and pH, these parameters need to be controlled (e.g. by electrical pacing) and reported.
